# Deep inspiration breath-hold radiation therapy in left-sided breast cancer patients: a single-institution retrospective dosimetric analysis of organs at risk doses

**DOI:** 10.1007/s00066-022-01998-z

**Published:** 2022-09-08

**Authors:** Jule Wolf, Sabine Stoller, Jördis Lübke, Thomas Rothe, Marco Serpa, Jutta Scholber, Constantinos Zamboglou, Eleni Gkika, Dimos Baltas, Ingolf Juhasz-Böss, Vivek Verma, David Krug, Anca-Ligia Grosu, Nils H. Nicolay, Tanja Sprave

**Affiliations:** 1 Department of Radiation Oncology, University Hospital of Freiburg, Robert-Koch-Str. 3, 79106 Freiburg, Germany; 2 German Cancer Consortium (DKTK) Partner Site Freiburg, German Cancer Research Center (dkfz), Neuenheimer Feld 280, 69120 Heidelberg, Germany; 3 Department of Obstetrics and Gynecology, Medical Center, University of Freiburg, Freiburg, Germany; 4 Department of Radiation Oncology, MD Anderson Cancer Center, Houston, TX USA; 5 Department of Radiation Oncology, University Hospital Schleswig-Holstein, Arnold-Heller-Str. 3, 24105 Kiel, Germany; 6 Department of Molecular and Radiation Oncology, German Cancer Research Center (dkfz), Neuenheimer Feld 280, 69120 Heidelberg, Germany

**Keywords:** Left-sided, Cardiac-sparing, Breast cancer, Deep inspiration breath-hold radiation therapy, Heart toxicity

## Abstract

**Background:**

Radiotherapy can induce cardiac injury in left-sided breast cancer cases. Cardiac-sparing irradiation using the deep inspiration breath-hold (DIBH) technique can achieve substantial dose reduction to vulnerable cardiac substructures compared with free breathing (FB). This study evaluated the dosimetric differences between both techniques at a single institution.

**Methods:**

From 2017 to 2019, 130 patients with left-sided breast cancer underwent breast-conserving surgery (BCS; *n* = 121, 93.1%) or mastectomy (ME; *n* = 9, 6.9%) along with axillary lymph node staging (*n* = 105, 80.8%), followed by adjuvant irradiation in DIBH technique; adjuvant systemic therapy was included if applicable. 106 (81.5%) patients received conventional and 24 (18.5%) hypofractionated irradiation. Additionally, 12 patients received regional nodal irradiation. Computed tomography (CT) scans in FB and DIBH position were performed for all patients. Intrafractional 3D position monitoring of the patient surface in deep inspiration and breath gating was performed using Sentinel and Catalyst HD 3D surface scanning systems (C-RAD, Catalyst, C‑RAD AB, Uppsala, Sweden). Individual coaching and determination of breathing amplitude during the radiation planning CT was performed. Three-dimensional treatment planning was performed using standard tangential treatment portals (6 or 18 MV). The delineation of cardiac structures and both lungs was done in both the FB and the DIBH scan.

**Results:**

All dosimetric parameters for cardiac structures were significantly reduced (*p* < 0.01 for all). The mean heart dose (Dmean) in the DIBH group was 1.3 Gy (range 0.5–3.6) vs. 2.2 Gy (range 0.9–8.8) in the FB group (*p* < 0.001). The Dmean for the left ventricle (LV) in DIBH was 1.5 Gy (range 0.6–4.5), as compared to 2.8 Gy (1.1–9.5) with FB (*p* < 0.001). The parameters for LV (V10 Gy, V15 Gy, V20 Gy, V23 Gy, V25 Gy, V30 Gy) were reduced by about 100% (*p* < 0.001). The LAD Dmean in the DIBH group was 4.1 Gy (range 1.2–33.3) and 14.3 Gy (range 2.4–37.5) in the FB group (*p* < 0.001). The median values for LAD such as V15 Gy, V20 Gy, V25 Gy, V30 Gy, and V40 Gy decreased by roughly 100% (*p* < 0.001). An increasing volume of left lung in the DIBH position resulted in dose sparing of cardiac structures.

**Conclusion:**

For all ascertained dosimetric parameters, a significant dose reduction could be achieved in DIBH technique.

**Supplementary Information:**

The online version of this article (10.1007/s00066-022-01998-z) contains supplementary material, which is available to authorized users.

## Introduction

Incidental irradiation of the heart for left-sided breast cancer increases the rate of subsequent ischemic cardiac events [1]. It has been suggested that the mean heart dose correlates linearly with a relative increase in cardiac events of 7.4% without a threshold [2]. Radiation-induced cardiac impairment results from damage to the micro- and macrovasculature [3, 4]. Dose-dependent vulnerability of the entire left ventricle and all coronary segments justifies rigorous dose reduction [5–8]. This dose-dependent rise occurs after a few years and persists for at least two decades [2]. Notably, preexisting cardiac risk factors increase the absolute risk caused by radiation therapy (RT) [2].

The aggregated cardiac toxicity after multimodality therapy consisting of chemotherapy and RT has not been well studied [9]. Higher doses of anthracyclines combined with higher dose volumes of cardiac irradiation are associated with an increased risk of cardiac events [10]. However, in selected non-high-risk cardiac patients, the multimodal approach appears relatively safe [11].

Deep inspiration breath-hold technique (DIBH) in the supine position is a commonly used heart-sparing approach for radiotherapy [12]. DIBH can be performed by tangential 3D conformal radiotherapy (3DRT) or rotational/multiangle intensity-modulated radiotherapy (IMRT/VMAT) [13]. Alternatively, in selected patients with a low-risk profile, partial breast irradiation can be performed using external beam RT [14, 15], brachytherapy [16–18], or intraoperative radiation therapy (IORT) alone [19, 20]. DIBH-based RT allows a reproducible cardiac shift from the irradiation field, resulting in substantial dose reduction to cardiac structures [12, 13].

In the absence of published data from randomized trials of DIBH vs. free breathing (FB) RT in the supine position, reporting institutional experiences is necessary. The goal of this single-institutional retrospective study was to compare dosimetric outcomes between DIBH and FB for left-sided breast cancer patients.

## Materials and methods

### Patient selection and treatment planning

**Fig. 1:**
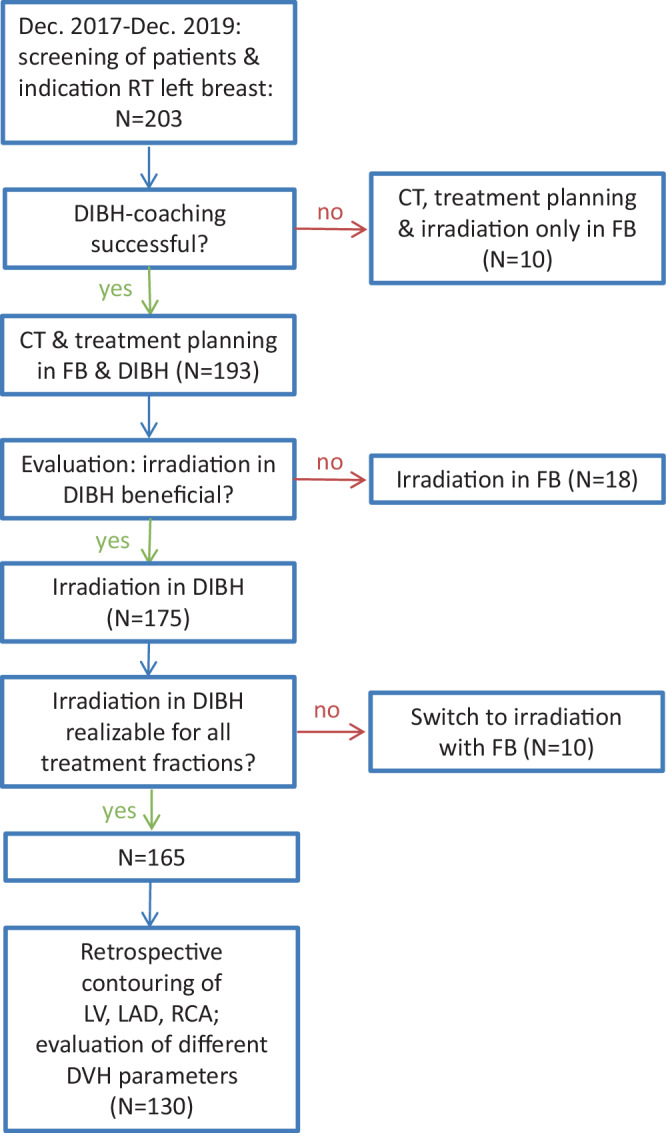
Flow chart of screening and inclusion procedures for this analysis. *DIBH* deep inspiration breath-hold, *CT* computed tomography, *FB* free breathing, *LV* left ventricle, *LAD* left anterior descending artery, *RCA* right coronary artery, *DVH* dose–volume histogram

From December 2017 to December 2019, 203 patients with left-sided or bilateral breast cancer were screened for irradiation in DIBH technique and 130 patients were included in this analysis (Fig. 1). Ten participants were not able to comply with the requirements of the DIBH technique, the other 193 patients received CT scans in FB and DIBH (Fig. 1). After the DIBH vs. FB plan comparison before starting radiotherapy, 18 patients did not show any dosimetric benefit for cardiac structures, so FB RT was performed for them. No reasons could be identified beforehand; however, the most common reasons turned out to be thoracic anatomy, respiratory depth, or patient compliance. Another 36 DIBH RT patients were excluded from evaluation due to technical difficulties related to retroactively contouring cardiac structures. As such, for the purposes of this study, contouring and evaluation of the plans was only possible for 130 patients.

Institutional criteria for patient selection for a tumor bed boost included patients with breast-conserving surgery (BCS), premenopausal status, or postmenopausal status in addition to the following risk factors: tumor size ≥ 2 cm, extensive intraductal component, grade 3 disease, HER2-positive disease, and triple-negative breast cancer (TNBC).

BCS or mastectomy with sentinel lymph node excision or axillary nodal dissection was performed according to institutional protocols. Neoadjuvant or adjuvant chemotherapy as well as endocrine therapy was administered based on the currently accepted guidelines and individual recommendations of the interdisciplinary oncological board.

**Fig. 2:**
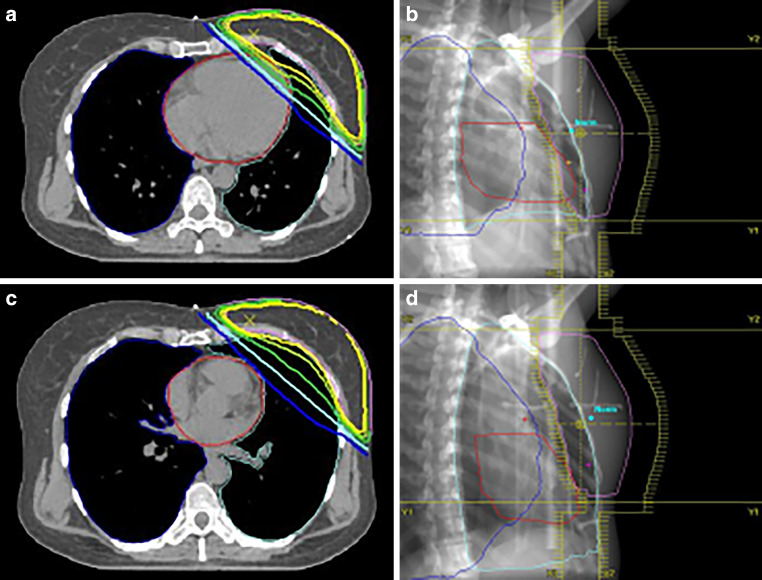
Isodose distribution in FB and DIBH plans for the same patient. **a** Axial scan in FB; **b** oblique reconstruction with visualization of tangential irradiation fields including the adaptations of the multileaf collimators “beam eye view,” 3DRT, 6 MV in FB; **c** axial scan in DIBH; **d** oblique reconstruction with visualization of tangential irradiation fields including the adapted of the multileaf collimators “beam eye view,” 3DRT, 6 MV in DIBH. *3DRT* 3D conformal radiotherapy, *DIBH* deep inspiration breath-hold, *FB* free breathing

All patients were coached on the DIBH technique in the CT room using a surface image-guided RT (SGRT) system (C-RAD, Catalyst, C‑RAD AB, Uppsala, Sweden). The patients were asked to take a deep breath and hold it for a duration of 20 s, and the width of the gating window was set to 5 mm. The patients received CT scans (Brilliance, CT Big Bore, Philips, Cleveland, OH) in FB and DIBH with a slice thickness of 2 mm. CT-based three-dimensional treatment planning (Oncentra MasterPlan, Nucletron, Veenendaal, the Netherlands, and/or Eclipse™ planning systems, Varian Medical Systems, Palo Alto, CA, USA) was performed using standard tangential treatment portals (6 or 18 MV; Synergy; Elekta, Crawley, United Kingdom; Fig. 2).

Subsequently, after completion of wound healing, adjuvant whole-breast irradiation (WBI) or thoracic wall RT was delivered according to standardized institutional protocols, which included hypofractionation (40.05 Gy in 15 fractions), conventional fractionation (50.0–50.4 Gy in 25–28 fractions), or with simultaneous integrated boost (58.8–61.6 Gy in 25–28 fractions). For indicated irradiation of lymph nodes (supraclavicular, axillary, internal mammary [IM]), a fractional dose of 1.7–1.8 Gy was used.

The institutional cardiac constraint was based on the following constraint of the DEGRO breast cancer expert panel: mean cardiac dose < 2.5 Gy [13]. An individualized decision was always made between heart doses and optimal target volume coverage, especially in the case of IM lymph node irradiation.

### Volume delineation and dosimetric comparison

For the planned dosimetric evaluation, the following cardiac structures of interest were retrospectively delineated: left ventricle (LV), left anterior descending artery (LAD), and right coronary artery (RCA). The delineation was done in both the FB and the DIBH scan according to the RTOG recommendations and the atlas by Feng et al. [21]. DVH parameters were then assigned for left and right lung (volume, Dmean, D50%, Dmax, V5 Gy, V20 Gy, D20%, D30%), left ventricle (volume, Dmean, D50%, Dmax, D2%, V5 Gy, V10 Gy, V15 Gy, V20 Gy, V23 Gy, V25 Gy, V30 Gy, V40 Gy), LAD and RCA (Dmean, D50%, Dmax, D2%, V5 Gy, V10 Gy, V15 Gy, V20 Gy, V25 Gy, V30 Gy, V40 Gy), and heart (volume, Dmean, D50%, Dmax, D2%, V5 Gy, V10 Gy, V15 Gy, V20 Gy, V25 Gy, V30 Gy, V40 Gy).

### Statistical analysis

Data are reported as a mean, median (range), and frequencies. For all dosimetric parameters, median values and their corresponding ranges as well as the relative dose reduction were determined. DVH parameters of the FB vs. DIBH plans were compared using either a paired *t*-test or a Wilcoxon signed-rank test. For evaluation of the impact of metric variables on dosimetric parameters (such as volumes of both lungs, heart, and cardiac subvolumes), univariate and multivariate analysis was performed using linear regression. Parameters that exhibited a *p*-value < 0.1 in univariate analysis were included in multivariate analysis.

*P*-values < 0.05 were considered statistically significant. Analysis was performed using SPSS version 27 (IBM, Armonk, NY, USA) and Microsoft Office Excel 2016 (Microsoft Corp. Redmond, WA, USA).

## Results

Altogether, 130 patients with 260 CT scans were analyzed (Fig. 1). Table 1 displays the treatment characteristics of this population. Most patients had T1 disease (66.2%, *n* = 86) and were node negative (87.6%, *n* = 114). Twelve participants had simultaneous RT of the regional lymph nodes: ten received RT of the ipsilateral supraclavicular and axillary lymph nodes and two additional IM. The vast majority of patients underwent breast-conserving surgery (93.1%, *n* = 121). After adjuvant RT, no locoregional recurrences were observed at a median follow-up of 4 months.

**Table 1:** Treatment characteristics of patients treated using the deep inspiration breath-hold technique for whole-breast or thoracic wall irradiation in our institution between 2017 and 2019 (*n* = 130)

Total patients: *n* = 130	*n*	%
*Radiotherapy*		
Conventional fractionation	106	81.5
Hypofractionation	24	18.5
SIB	43	33.1
SEB	2	1.5
IORT	49	37.7
3DRT	130	100
*Lymph node irradiation*		
Yes	12	9.2
No	118	90.8

*3DRT* 3D-conformal radiotherapy, *IORT* intraoperative radiotherapy, *SEB* sequential boost, *SIB* simultaneous integrated boost

### Heart: LV, LAD, and RCA

The DVH parameters for heart structures and both lungs are summarized in Table 2 and Supplementary Table 1. All ascertained dosimetric parameters for all listed cardiac structures were significantly reduced (*p* < 0.01 for all) in the DIBH position (Table 2).

**Table 2:** Comparison of absolute mean values (ranges) of DVH parameters for whole heart, LV, LAD, and RCA and relative changes (%) between DIBH and FB techniques using two-sided significances of changes in distributions of these measures

DVH parameter	FB	DIBH	Reduction [%]	*p*-value
*Heart*				
Volume [ccm]	602.1 (378.2–891.8)	555.6 (318.9–884.9)	−7.7	< 0.001
Dmean [Gy]	2.2 (0.9–8.8)	1.3 (0.5–3.6)	−41.0	< 0.001
D50% [Gy]	1.3 (0.5–2.6)	1.0 (0.4–2.0)	−18.3	< 0.001
Dmax [Gy]	46.1 (12.9–59.4)	21.2 (3.6–56.0)	−54.1	< 0.001
D2% [Gy]	14.4 (2.4–48.2)	3.6 (1.3–37.9)	−74.9	< 0.001
V5 Gy [%]	4.8 (0.1–22.6)	0.7 (0.0–10.0)	−84.7	< 0.001
V10 Gy [%]	2.8 (0.0–13.0)	0.1 (0.0–6.3)	−97.8	< 0.001
V15 Gy [%]	2.0 (0.0–10.4)	0.0 (0.0–5.1)	−100.0	< 0.001
V20 Gy [%]	1.6 (0.0–8.7)	0.0 (0.0–4.2)	−100.0	< 0.001
V25 Gy [%]	1.2 (0.0–7.6)	0.0 (0.0–3.5)	−100.0	< 0.001
V30 Gy [%]	0.9 (0.0–6.7)	0.0 (0.0–2.9)	−100.0	< 0.001
V40 Gy [%]	0.2 (0.0–4.9)	0.0 (0.0–1.7)	−100.0	< 0.001
*Left ventricle*				
Volume [ccm]	168.5 (82.1–308.1)	156.7 (69.2–290.8)	−7.0	< 0.001
Dmean [Gy ]	2.8 (1.1–9.5)	1.5 (0.6–4.5)	−46.6	< 0.001
D50% [Gy]	1.8 (0.9–3.5)	1.3 (0.5–2.5)	−26.8	< 0.001
Dmax [Gy]	43.5 (7.7–57.9)	11.2 (2.1–54.1)	−74.2	< 0.001
D2% [Gy]	16.7 (2.5–50.5)	3.3 (1.2–39.5)	−80.2	< 0.001
V5 Gy [%]	7.3 (0.0–36.0)	0.2 (0.0–15.6)	−96.7	< 0.001
V10 Gy [%]	3.6 (0.0–22.2)	0.0 (0.0–9.8)	−100.0	< 0.001
V15 Gy [%]	2.4 (0.0–19.3)	0.0 (0.0–7.2)	−100.0	< 0.001
V20 Gy [%]	1.6 (0.0–17.3)	0.0 (0.0–5.2)	−100.0	< 0.001
V23 Gy [%]	1.2 (0.0–16.1)	0.0 (0.0–4.7)	−100.0	< 0.001
V25 Gy [%]	1.0 (0.0–15.5)	0.0 (0.0–4.3)	−100.0	< 0.001
V30 Gy [%]	0.6 (0.0–13.7)	0.0 (0.0–3.5)	−100.0	< 0.001
V40 Gy [%]	0.0 (0.0–8.0)	0.0 (0.0–1.9)	−100.0	< 0.01
*LAD*				
Dmean [Gy]	14.3 (2.4–37.5)	4.1 (1.2–33.3)	−71.3	< 0.001
D50% [Gy]	9.8 (2.1–45.4)	3.7 (1.2–44.4)	−62.5	< 0.001
Dmax [Gy]	43.4 (5.2–59.1)	16.3 (2.1–55.2)	−62.5	< 0.001
D2% [Gy]	38.7 (4.0–57.4)	8.9 (1.8–51.1)	−77.0	< 0.001
V5 Gy [%]	72.1 (0.0–100.0)	20.8 (0.0–97.9)	−71,1	< 0.001
V10 Gy [%]	49.8 (0.0–96.5)	0.8 (0.0–84.3)	−98.4	< 0.001
V15 Gy [%]	37.9 (0.0–92.5)	0.0 (0.0–81.3)	−100.0	< 0.001
V20 Gy [%]	28.1 (0.0–85.7)	0.0 (0.0–79.8)	−100.0	< 0.001
V25 Gy [%]	22.1 (0.0–80.8)	0.0 (0.0–75.8)	−100.0	< 0.001
V30 Gy [%]	14.4 (0.0–79.0)	0.0 (0.0–72.3)	−100.0	< 0.001
V40 Gy [%]	0.51 (0.0–70.2)	0.0 (0.0–60.0)	−100.0	< 0.001
*RCA*				
Dmean [Gy]	1.2 (0.5–2.5)	1.0 (0.4–1.9)	−21.0	< 0.001
D50% [Gy]	1.2 (0.5–2.5)	1.0 (0.4–1.8)	−19.0	< 0.001
Dmax [Gy]	1.7 (0.8–3.9)	1.5 (0.6–2.7)	−14.1	< 0.001
D2% [Gy]	1.6 (0.7–3.5)	1.4 (0.6–2.5)	−14.6	< 0.001
V5 Gy [%]	0.0 (0.0–0.0)	0.0 (0.0–0.0)	0.0	–
V10 Gy [%]	0.0 (0.0–0.0)	0.0 (0.0–0.0)	0.0	–
V15 Gy [%]	0.0 (0.0–0.0)	0.0 (0.0–0.0)	0.0	–
V20 Gy [%]	0.0 (0.0–0.0)	0.0 (0.0–0.0)	0.0	–
V25 Gy [%]	0.0 (0.0–0.0)	0.0 (0.0–0.0)	0.0	–
V30 Gy [%]	0.0 (0.0–0.0)	0.0 (0.0–0.0)	0.0	–
V40 Gy [%]	0.0 (0.0–0.0)	0.0 (0.0–0.0)	0.0	–

*DIBH* deep inspiration breath-hold, *DVH* dose–volume histogram, *FB* free breathing, *LAD* left anterior descending artery, *LV* left ventricle, *RCA* right coronary artery

D_mean_ Gy, D 50% Gy, D_max_ Gy, D 2% Gy, V5 Gy %, V10Gy %, V15Gy %, V20Gy %, V23Gy %, V25Gy %, V30Gy %, V40Gy %

The mean heart dose (Dmean) in the DIBH group was 1.3 Gy (range 0.5–3.6) vs. 2.2 Gy (range 0.9–8.8) in the FB group. The mean heart Dmax dose in the DIBH group was reduced > 50% in comparison to the FB group. The mean values of V15 Gy, V20 Gy, V25 Gy, V30 Gy, and V40 Gy for heart in the DIBH cohort could be decreased by approximately 100% in comparison to the FB group.

**Fig. 3:**
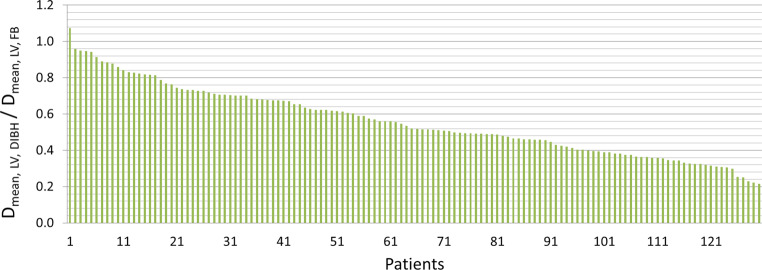
Relative ratio of Dmean LV in DIBH to FB position (y-axis) in the entire cohort (x-axis, *n* = 130). *DIBH* deep inspiration breath-hold, *FB* free breathing, *LV* left ventricle

The Dmean for LV in the DIBH technique was 1.5 Gy (range 0.6–4.5) and 2.8 Gy (1.1–9.5) with FB. In half of the patients, the LV Dmean was reduced by about 50% (Fig. 3).

The listed mean dosimetric values specifically for LV (V10 Gy, V15 Gy, V20 Gy, V23 Gy, V25 Gy, V30 Gy, V40 Gy) were reduced by approximately 100%.

**Fig. 4:**
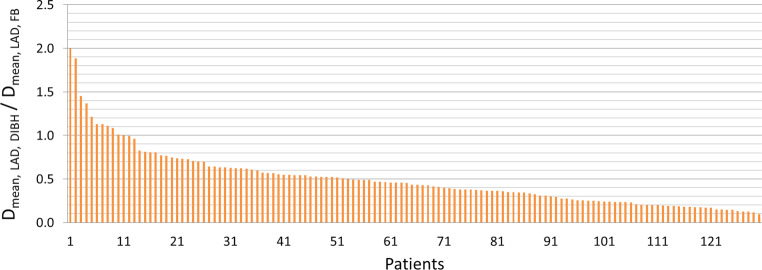
Relative ratio of Dmean LAD in DIBH to FB position (y-axis) in the entire cohort (x-axis, *n* = 130). *DIBH* deep inspiration breath-hold, *FB* free breathing, *LAD* left anterior descending artery

The LAD Dmean in the DIBH group was on average 4.1 Gy (range 1.2–33.3) and 14.3 Gy (range 2.4–37.5) in the FB group. In half of the patients, the LAD Dmean was reduced by approximately 50% (Fig. 4). Consistently, the median values for LAD such as V15 Gy, V20 Gy, V23 Gy, V25 Gy, V30 Gy, and V40 Gy decreased by almost 100%.

The same trend was observed when considering the RCA Dmean: 1.0 Gy (range 0.4–1.9) in the DIBH group vs. 1.2 Gy (range 0.5–2.5) in FB group.

### Lungs

With DIBH, the Dmean parameters of the left and right lungs were reduced by approximately 7% and 12%, respectively. The V5 Gy for the left lung in the DIBH group was 24.0% (range 11.2–44.3) vs. 24.9% (range 10.4–53.0) in the FB group. More pronounced was the observed V20 Gy reduction for the left lung with DIBH; on average 10.4% (range 0–22.9) vs. 12.1% (range 1.9–31.6). The right lung Dmean in the DIBH group was reduced by approximately 12% in comparison to the FB group.

The results of univariate and multivariate analyses are reported in Table 3. The left lung volume in the DIBH position was the independent variable in multivariate analysis (Table 3). When considering relevant dosimetric parameters based on the DEGRO breast cancer expert panel recommendations [13] such as heart Dmean, LV Dmean, LV V5 Gy, LAD Dmean, and LAD V30 Gy, only the left lung volume in the DIBH technique remained an independent predictor in multivariate analysis. Increasing left lung volumes (in DIBH) showed a dose-sparing effect (Table 3) only for LV V23 Gy and LAD V40 Gy and in the univariate analysis.

**Table 3:** Univariate and multivariate analysis for selected DVH parameters of heart structures in DIBH position

DVH parameter	Univariate analysis	*p*-value	Multivariate analysis	*p*-value
*Heart*				
Dmean [Gy]	Left lung volume DIBH	< 0.001	Left lung volume DIBH	0.002
	Left lung volume FB	< 0.001		
	Right lung volume DIBH	< 0.001		
	Right lung volume FB	< 0.001		
V20 Gy [%]	Left lung volume DIBH	< 0.001	Left lung volume DIBH	< 0.001
	Left lung volume FB	0.016		
	Right lung volume DIBH	0.001		
	Heart volume DIBH	0.034		
*Left ventricle*				
Dmean [Gy]	Left lung volume DIBH	< 0.001	Left lung volume DIBH	< 0.001
	Left lung volume FB	< 0.001		
	Right lung volume DIBH	< 0.001		
V5 Gy [%]	Left lung volume DIBH	< 0.001	Left lung volume DIBH	0.008
	Left lung volume FB	< 0.001		
	Right lung volume DIBH	0.003		
	Right lung volume FB	< 0.001		
V23 Gy [%]	Left lung volume DIBH	0.001	–	–
*LAD*				
Dmean [Gy]	Left lung volume DIBH	< 0.001	Left lung volume DIBH	0.022
	Left lung volume FB	0.001		
	Right lung volume DIBH	< 0.001		
	Right lung volume FB	0.01		
V30 Gy [%]	Left lung volume DIBH	< 0.001	Left lung volume DIBH	< 0.001
	Left lung volume FB	0.02		
	Right lung volume DIBH	< 0.001		
V40 Gy [%]	Left lung volume DIBH	0.003	–	–

*DIBH* deep inspiration breath-hold, *DVH* dose–volume histogram, *FB* free breathing, *LAD* left anterior descending artery, *LV* left ventricle

## Discussion

Various studies have shown a benefit of DIBH in tangential 3DRT technique in regards to significant dose reduction of cardiac structures [22–25]. In line with previous studies, this single-institutional retrospective comparison demonstrates that the DIBH technique achieved a significant dose reduction for most analyzed dosimetric parameters with acceptable patient compliance.

All analyzed DVH parameters for all cardiac structures could be significantly reduced (*p* < 0.01 for all) in the DIBH position (Table 2). The heart Dmean in the DIBH group could be reduced by about > 40% in comparison to the FB group. Comparable with our results, Simonetto et al. showed reduction of mean heart doses by 35% (interquartile range 23–46%) in DIBH in comparison to FB [26]. The mean heart parameters for V15–V40 Gy in the DIBH cohort could be decreased by almost 100% in comparison to the FB group. Additionally, the LV Dmean with DIBH was 1.5 Gy (range 0.6–4.5) in contrast to 2.8 Gy (1.1–9.5) with FB. In half of the patients, LV Dmean was reduced by about 50% (Fig. 3).

The LAD Dmean in the DIBH group was 4.1 Gy (range 1.2–33.3) and 14.3 Gy (range 2.4–37.5) in the FB group. In contrast, Joo et al. demonstrated significantly superior Dmean reduction to the LAD, from 4079.1 cGy in FB to 2368.9 cGy in DIBH (*p* < 0.001), in comparison with our results [27]. Another study showed the mean LAD dose was 1.5 Gy with DIBH vs. 19.8 Gy with FB (*p* < 0.001), which may be more comparable with our values [28]. In half of the patients in our study, Dmean LAD was reduced by approximately 50% (Fig. 4).

Of note, the cardiopulmonary dose sparing for V50 Gy using the relative seriality model diminishes the likelihood of pneumonitis and cardiac mortality [29]. Our study demonstrates dose sparing in DIBH position by 100% for V40 Gy in heart (Table 2). Herein, long-term follow-up clinical data were not assessed. In breast cancer survivors, a radiation therapy-related mortality risk may persist for two decades and possibly even increase in the third [30]. In light of this, recent models show that in the presence of radiation-induced cardiac mortality, the mean expected years of life lost appears to be lower at 0.07 in DIBH cohorts vs. 0.11 in FB [26]. This DIBH effect was even more prominent in patients with high mean cardiac doses in FB (0.09 years for doses > 3 Gy vs. 0.02 years for doses < 1.5 Gy) [26]. Additionally, preexisting cardiovascular risk factors at baseline such as diabetes and smoking had a substantial impact on the 10-year cumulative risk for cardiovascular disease, which was not completely diminished using DIBH [31]. Unexpectedly, Jimenez et al. found that using modern adjuvant RT in ≥ 60-year-old women with right- or left-sided hormone receptor-positive early breast cancer is not associated with an increased risk of cardiac mortality within 10 years of RT [32]. Unfortunately, the authors did not provide any information on DIBH use [32].

DIBH decreased most DVH values for the left lung, in particular the Dmean by 7.3% and V20 Gy by 14.0% (Supplementary Table 1). Our results are similar to the study by Oechsner et al., wherein DIBH reduced the left lung Dmean by −19 ± 9% and the relative V20 Gy by −24 ± 10% [33]. Additionally, in our study, the left lung volume in DIBH was an independent predictor of reduced cardiac DVH parameters in multivariate analysis (Table 3), which is consistent with the results of the aforementioned study [33].

Accelerated partial breast irradiation is a possible alternative to whole-breast irradiation for selected women with a low-risk profile, as it can shorten treatment time and reduce radiation exposure to surrounding tissue [14, 15, 34]. A relevant prospective study showed that interstitial multicatheter APBI achieves equivalent local control compared to whole-breast RT [16]. The use of interstitial multicatheters may reduce the risk of late skin side effects [35]. However, when considering relevant dosimetric outcomes for LAD and left lung, the mean values are comparable to DIBH [36, 37]. However, the choice of APBI technique must be tailored to the location of tumor, treatment goals, and patient preferences [38].

Limitations of our study are, among others, the heterogeneity of delivered doses and treatment planning techniques and the lack of adjustment for existing cardiac risk factors. No adjustment was made for different dose/fractionation schemes. As the left lung volume was the only predictor of cardiac dose reduction, individual differences in respiratory fitness may be a major factor. There was no adjustment for this in our study. Optimal coaching of patients for DIBH may improve results further. We set the gating window to 5 mm to ensure robust radiation treatment and patient compliance and to reduce treatment time. However, with a smaller gating window, cardiac sparing might have been improved. Except for patients on trastuzumab, regular cardiologic follow-up was not conducted. Thus, the clinical outcome and relevance of cardiac dose reduction remains to be analyzed. Additionally, the screening of baseline cardiac risk factors and risk-adapted cardiological and radiotherapeutic follow-up should be adapted for the vulnerable risk groups. About 10% of patients derived little or no benefit from DIBH in terms of Dmean LAD. This may be related to suboptimal compliance with DIBH or individual anatomic circumstances, which is why we performed CT scans in DIBH and free breathing for all patients. The data can only be used for DIBH-radiation in breast cancer without elective lymph node irradiation, as the latter group is not adequately represented in this retrospective analysis.

## Supplementary Information

Supplement: Table 1: Comparison of selected DVH parameters of both lungs in DIBH and FB techniques. Comparison of absolute mean values (ranges) of DVH parameters for both lungs and relative changes in percent between DIBH and FB techniques using two-sided significances of changes in distributions of these measures

Supplement: Table 2: Univariate and multivariate analysis for selected DVH parameters from heart structures in DIBH position. *DIBH* deep inspiration breath-hold; *DVH* dose–volume histogram; *FB* free breathing; *LAD* left anterior descending artery; *LV* left ventricle; *RCA* right coronary artery

## Abbreviations

ALND: Axillary lymph node dissection
APBI: Accelerated partial breast irradiation
BCS: Breast conservation surgery
CI: Confidence interval
DIBH: Deep inspiration breath-hold
3DRT: 3D-Conformal radiotherapy
DVH: Dose–volume histogram
ER: Estrogen receptor
FB: Free breathing
HER2: Human epidermal growth factor receptor 2
HT: Hormonal therapy
IM: Internal mammary
IMRT: Intensity-modulated radiotherapy
IORT: Intraoperative radiotherapy
LAD: Left anterior descending artery
LV: Left ventricle
MV: Megavolt
OS: Overall survival
RCA: Right coronary artery
RT: Radiotherapy
SEB: Sequential boost
SGRT: Surface image-guided radiotherapy
SIB: Simultaneous integrated boost
SLND: Sentinel lymph node dissection
TNBC: Triple-negative breast cancer
VMAT: Volumetric modulated arc therapy
WBI: Whole-breast irradiation
