# Supplementary material for: Deep inspiration breath-hold radiation therapy in left-sided breast cancer patients: a single-institution retrospective dosimetric analysis of organs at risk doses
Source: Strahlenther Onkol. 2022 Sep 8;199(4):379–88. doi: 10.1007/s00066-022-01998-z (PMC10033469; doi:10.1007/s00066-022-01998-z)
Supplement: Supplementary file 1 — Supplement: Table 1: Comparison of selected DVH parameters of both lungs in DIBH and FB techniques. Comparison of absolute mean values (ranges) of DVH parameters for both lungs and relative changes in percent between DIBH and FB techniques using two-sided significances of changes in distributions of these measures [file 66_2022_1998_MOESM1_ESM.docx]

Supplement

Table 1

| **DVH parameter** | **FB** | **DIBH** | **Reduction [%]** | **p-value** |
| --- | --- | --- | --- | --- |
| **Left Lung** |  |  |  |  |
| Volume [ccm] | 1333.7 (677.4-2260.9) | 2397.8 (1290.3-3216.4) | 79,8 | <0.001 |
| D mean [Gy] | 6.5 (2.2-15.6) | 6.0 (2.7-11.6) | -7,3 | <0.001 |
| D50% [Gy] | 1.7 (0.7-5.9) | 1.5 (0.7-3.8) | -11,3 | <0.001 |
| D max [Gy] | 51.1 (35.5-63.4) | 50.9 (37.8-61.9) | -0,3 | 0,231 |
| V5 Gy [%] | 24.9 (10.4-53.0) | 24.0 (11.2-44.3) | -0,0 | 0,001 |
| V20 Gy [%] | 12.1 (1.9-31.6) | 10.4 (0.0-22.9) | -14,0 | <0.001 |
| D20% [Gy] | 7.2 (2.4-38.0) | 6.6 (2.4-25.0) | -8,6 | <0.001 |
| D30% [Gy] | 3.7 (1.4-22.2) | 3.6 (1.4-11.5) | -4,0 | 0,001 |
|  |  |  |  |  |
| **Right Lung** |  |  |  |  |
| Volume [ccm] | 1624.7 (823.4-2362.8) | 2747.1 (1450.7-3538.9) | 69,1 | <0.001 |
| D mean [Gy] | 0.6 (0.2-6.8) | 0.5 (0.2-5.3) | -12,3 | <0.001 |
| D50% [Gy] | 0.5 (0.1-1.5) | 0.5 (0.2-1.1) | -13,0 | <0.001 |
| D max [Gy] | 2.5 (1.0-51.4) | 2.9 (1.0-51.4) | 16,2 | <0.001 |
| V5 Gy [%] | 0.0 (0.0-25.2) | 0.0 (0.0-19.7) | 0,0 | 0,484 |
| V20 Gy [%] | 0.0 (0.0-11.9) | 0.0 (0.0-8.7) | 0,0 | 0,593 |
| D20% [Gy] | 0.7 (0.2-7.6) | 0.6 (0.3-4.9) | -7,2 | <0.001 |
| D30% [Gy] | 0.6 (0.2-3.8) | 0.6 (0.3-2.7) | -8,0 | <0.001 |
