# Supplementary material for: Deep inspiration breath-hold radiation therapy in left-sided breast cancer patients: a single-institution retrospective dosimetric analysis of organs at risk doses
Source: Strahlenther Onkol. 2022 Sep 8;199(4):379–88. doi: 10.1007/s00066-022-01998-z (PMC10033469; doi:10.1007/s00066-022-01998-z)
Supplement: Supplementary file 2 — Supplement: Table 2: Univariate and multivariate analysis for selected DVH parameters from heart structures in DIBH position. DIBH deep inspiration breath-hold; DVH dose–volume histogram; FB free breathing; LAD left anterior descending artery; LV left ventricle; RCA right coronary artery [file 66_2022_1998_MOESM2_ESM.docx]

Supplement Table 2

| **DIBH** | | | | |
| --- | --- | --- | --- | --- |
| **DVH parameter** | **Univariate analysis** | **p-value** | **Multivariate analysis** | **p-value** |
|  |  |  |  |  |
| **Heart** |  |  |  |  |
|  |  |  |  |  |
| D50% [Gy] | Left lung volume DIBH | <0.001 |  |  |
|  | Left lung volume FB | <0.001 |  |  |
|  | Right lung volume DIBH | <0.001 |  |  |
|  | Right lung volume FB | <0.001 | Right lung volume FB | 0.04 |
|  |  |  |  |  |
| D max [Gy] | Lef ventricle volume DIBH | <0.001 | Lef ventricle volume DIBH | <0.001 |
|  | Left lung volume DIBH | <0.001 | Left lung volume DIBH | 0,006 |
|  | Left lung volume FB | <0.001 |  |  |
|  | Right lung volume DIBH | 0,002 | Right lung volume DIBH | 0,043 |
|  | Right lung volume FB | 0,002 |  |  |
|  |  |  |  |  |
| D2% [Gy] | Heart volume DIBH | 0,044 |  |  |
|  | Left lung volume DIBH | <0.001 |  |  |
|  | Left lung volume FB | 0,006 |  |  |
|  | Right lung volume DIBH | <0.001 |  |  |
|  |  |  |  |  |
| V5 Gy [%] | Left lung volume DIBH | <0.001 | Left lung volume DIBH | 0,039 |
|  | Left lung volume FB | <0.001 |  |  |
|  | Right lung volume DIBH | <0.001 |  |  |
|  | Right lung volume FB | <0.001 |  |  |
|  |  |  |  |  |
| V10 Gy [%] | Left lung volume DIBH | <0.001 | Left lung volume DIBH | 0,018 |
|  | Left lung volume FB | 0,002 |  |  |
|  | Right lung volume DIBH | <0.001 |  |  |
|  | Right lung volume FB | 0,037 |  |  |
|  |  |  |  |  |
| V15 Gy [%] | Left lung volume DIBH | <0.001 | Left lung volume DIBH | <0.001 |
|  | Left lung volume FB | 0,007 |  |  |
|  | Right lung volume DIBH | <0.001 | Right lung volume DIBH | 0,037 |
|  |  |  |  |  |
| V25 Gy [%] | Left lung volume DIBH | <0.001 | Left lung volume DIBH | <0.001 |
|  | Left lung volume FB | 0,039 | Left lung volume FB | 0,038 |
|  | Right lung volume DIBH | 0,002 | Right lung volume DIBH | 0,018 |
|  |  |  |  |  |
| V30 Gy [%] | Left lung volume DIBH | <0.001 | Left lung volume DIBH | <0.001 |
|  | Right lung volume DIBH | 0,008 | Right lung volume DIBH | 0,025 |
|  |  |  |  |  |
| V40 Gy [%] | Left lung volume DIBH | 0,007 |  |  |
|  |  |  |  |  |
| **Left Ventricle** |  |  |  |  |
|  |  |  |  |  |
| D50% [Gy] | Left lung volume DIBH | <0.001 | Left lung volume DIBH | 0.021 |
|  | Left lung volume FB | <0.001 |  |  |
|  | Right lung volume DIBH | <0.001 |  |  |
|  | Right lung volume FB | <0.001 | Right lung volume FB | 0.03 |
|  |  |  |  |  |
| D max [Gy] | Left lung volume DIBH | <0.001 | Left lung volume DIBH | <0.001 |
|  | Left lung volume FB | 0,002 |  |  |
|  | Right lung volume DIBH | 0,01 |  |  |
|  | Right lung volume FB | 0,017 |  |  |
|  |  |  |  |  |
| D2% [Gy] | Left lung volume DIBH | <0.001 | Left lung volume DIBH | <0.001 |
|  | Left lung volume FB | 0,023 |  |  |
|  | Right lung volume DIBH | 0,003 | Right lung volume DIBH | 0,005 |
|  |  |  |  |  |
| V10 Gy [%] | Left lung volume DIBH | <0.001 | Left lung volume DIBH | 0,002 |
|  | Left lung volume FB | <0.001 |  |  |
|  | Right lung volume DIBH | 0,003 |  |  |
|  | Right lung volume FB | <0.001 |  |  |
|  |  |  |  |  |
| V15 Gy [%] | Left lung volume DIBH | <0.001 | Left lung volume DIBH | <0.001 |
|  | Right lung volume DIBH | 0,017 | Right lung volume DIBH | 0,008 |
|  |  |  |  |  |
| V20 Gy [%] | Left lung volume DIBH | <0.001 |  |  |
|  |  |  |  |  |
| V25 Gy [%] | Left lung volume DIBH | 0,002 |  |  |
|  |  |  |  |  |
| V30 Gy [%] | Left lung volume DIBH | 0,001 |  |  |
|  |  |  |  |  |
| V40 Gy [%] | Right lung volume FB | 0,038 |  |  |
|  |  |  |  |  |
| **LAD** |  |  |  |  |
|  |  |  |  |  |
| D50% [Gy] | Left lung volume DIBH | <0.001 |  |  |
|  | Left lung volume FB | 0,001 |  |  |
|  | Right lung volume DIBH | <0.001 |  |  |
|  | Right lung volume FB | 0,012 |  |  |
|  |  |  |  |  |
| D max [Gy] | Left lung volume DIBH | <0.001 | Left lung volume DIBH | 0,006 |
|  | Left lung volume FB | 0,003 |  |  |
|  | Right lung volume DIBH | 0,001 |  |  |
|  | Right lung volume FB | 0,022 |  |  |
|  |  |  |  |  |
| D2% [Gy] | Left lung volume DIBH | <0.001 | Left lung volume DIBH | 0,013 |
|  | Left lung volume FB | 0,003 |  |  |
|  | Right lung volume DIBH | <0.001 |  |  |
|  | Right lung volume FB | 0,026 |  |  |
|  |  |  |  |  |
| V5 Gy [%] | Left lung volume DIBH | <0.001 | Left lung volume DIBH | 0.021 |
|  | Left lung volume FB | <0.001 |  |  |
|  | Right lung volume DIBH | <0.001 |  |  |
|  | Right lung volume FB | <0.001 |  |  |
|  |  |  |  |  |
| V10 Gy [%] | Left ventricle volume DIBH | 0,018 | Left ventricle volume DIBH | 0,008 |
|  | Left lung volume DIBH | <0.001 |  |  |
|  | Left lung volume FB | 0,001 |  |  |
|  | Right lung volume DIBH | <0.001 |  |  |
|  | Right lung volume FB | 0,004 |  |  |
|  |  |  |  |  |
| V15 Gy [%] | Left ventricle volume DIBH | 0,039 |  |  |
|  | Left lung volume DIBH | <0.001 |  |  |
|  | Left lung volume FB | 0,001 |  |  |
|  | Right lung volume DIBH | <0.001 |  |  |
|  | Right lung volume FB | 0,011 |  |  |
|  |  |  |  |  |
| V20 Gy [%] | Left lung volume DIBH | <0.001 |  |  |
|  | Left lung volume FB | <0.001 |  |  |
|  | Right lung volume DIBH | 0,01 |  |  |
|  | Right lung volume FB | <0.001 |  |  |
|  |  |  |  |  |
| V25 Gy [%] | Left lung volume DIBH | <0.001 | Left lung volume DIBH | <0.001 |
|  | Left lung volume FB | 0,006 |  |  |
|  | Right lung volume DIBH | <0.001 |  |  |
|  |  |  |  |  |
| **RCA** |  |  |  |  |
| D mean [Gy] | Left lung volume DIBH | <0.001 |  |  |
|  | Left lung volume FB | <0.001 |  |  |
|  | Right lung volume DIBH | <0.001 |  |  |
|  | Right lung volume FB | <0.001 |  |  |
|  |  |  |  |  |
| D50% [Gy] | Left lung volume DIBH | <0.001 |  |  |
|  | Left lung volume FB | <0.001 |  |  |
|  | Right lung volume DIBH | <0.001 |  |  |
|  | Right lung volume FB | <0.001 |  |  |
|  |  |  |  |  |
| D max [Gy] | Left lung volume DIBH | 0,006 |  |  |
|  | Left lung volume FB | <0.001 |  |  |
|  | Right lung volume DIBH | 0,002 |  |  |
|  | Right lung volume FB | <0.001 |  |  |
|  |  |  |  |  |
| D2% [Gy] | Left lung volume DIBH | 0,004 |  |  |
|  | Left lung volume FB | <0.001 |  |  |
|  | Right lung volume DIBH | 0,002 |  |  |
|  | Right lung volume FB | <0.001 |  |  |
|  |  |  |  |  |
|  |  |  |  |  |
